# The ecology of zebra finch song and its implications for vocal communication in multi-level societies

**DOI:** 10.1098/rstb.2023.0191

**Published:** 2024-06-23

**Authors:** Hugo Loning, Simon C. Griffith, Marc Naguib

**Affiliations:** ^1^ Behavioural Ecology Group, Wageningen University & Research, 6708 WD, The Netherlands; ^2^ School of Natural Sciences, Macquarie University, Sydney, New South Wales 2109, Australia; ^3^ School of Biological, Earth & Environmental Sciences, University of New South Wales, Sydney, New South Wales 2052, Australia

**Keywords:** spatial organization, social hotspot, birdsong, low-amplitude vocalizations, song ecology, *Taeniopygia castanotis*

## Abstract

Acoustic signalling is crucial in affecting movements and in social interactions. In species with dynamic social structures, such as multi-level societies, acoustic signals can provide a key mechanism allowing individuals to identify and find or avoid each other and to exchange information. Yet, if the spacing between individuals regularly exceeds the maximum signalling range, the relation between movements and signals becomes more complex. As the best-studied songbird in captivity, the zebra finch (*Taeniopygia castanotis*) is a species with individually distinct songs that are audible over just a few metres and a widely ranging dynamic multi-level social organization in the wild, raising questions on the actual role of its song in social cohesion and coordination. Here, we provide an overview of birdsong in social organizations (networks) and use the ecology of the zebra finch and male song to discuss how singing can facilitate social cohesion and coordination in species where the signal range is very short. We raise the question of the extent to which zebra finches are a representative species to understand the function of song in communication, and we broaden current views on the function of birdsong and its individual signature.

This article is part of the theme issue 'The power of sound: unravelling how acoustic communication shapes group dynamics'.

## 1. Birdsong and its role in spatial organization

Animals communicate in amazingly diverse and often complex ways, raising many questions on the mechanisms and functions of these signalling systems as well as on their ontogeny and evolution. Birdsong has been among the best-studied model systems in animal communication, providing insights into many facets of animal physiology, behaviour, ecology and evolution in more general terms [1,2]. As a long-range signal, birdsong plays an important role in the wider social organization of individuals as it affects the movements of others by attracting or repelling them, thus driving settlement patterns of territorial animals as well as movement trajectories by others [3,4]. Understanding the function of song in this wider social organization requires understanding the communication distance of the signals and the context of singing, as well as the effects on receivers under different ecological and social conditions.

Much of the extensive literature on the function of birdsong is focused on territorial species in temperate regions of the Northern Hemisphere (i.e. Europe and North America), with much research being based on particularly well-studied species from this region, such as tits, thrushes, starlings, flycatchers and emberizid sparrows [5–10]. This has created our foundational understanding of birdsong communication systems and the function of birdsong in mate attraction and territory defence [2]. Yet, the general ideas on the functions of birdsong derived from these systems may not fully represent the wide variety of singing contexts in these and other songbird species across the globe living under different social and ecological conditions. For example, most species in the temperate zones sing loudly. Birdsong and often acoustic communication in general thus are framed as a classic form of long-range communication [11–13]. Indeed, many studies have addressed the idea that vocalizations evolved to transmit far with the least degradation, known as the acoustic adaptation hypothesis [14]. Only more recently has there been an increase in attention to the less conspicuous soft song, often termed ‘quiet song’, which is often produced during moments of high arousal such as during short-range territorial disputes or courtship [15–20].

In a similar way, in the songbird species that are historically well-studied in the wild with respect to the function of song (as listed above), males are typically the singing sex. Yet, it was discovered recently in a more global analysis that female song is the ancestral state in songbirds, with about 65% of songbird species having a song in both sexes [21,22]. So, in addition to studying why males sing, one also could consider why females in certain species, in an evolutionary sense, ceased singing. Furthermore, it raises the question of to what extent birdsong may also function beyond the classical interpretation as a primarily sexually selected trait for mate choice and territory defence. For example, a more nuanced perspective may consider that song could also underlie strong sexual selection after mate choice to optimize reproduction with the existing partner [23,24]. The presence of song in both sexes in many avian species suggests that it may additionally be a socially selected signal [25–27] used also in competition for non-sexual resources [28] or in cooperation [23]. As this emancipation of the classical birdsong literature progresses and biases are increasingly addressed, it is likely that we will also encounter additional functions of song [29]. A strength of birdsong as a model communication system is the ability to study mechanisms and immediate functions under controlled laboratory conditions as well as in the field [1]. Yet, with a few exceptions, such as the starling (*Sturnus vulgaris*), great tit (*Parus major*), song sparrow (*Melospiza melodia*) and swamp sparrow (*M. georgiana*), research in the laboratory has focussed on species that are less well studied in the wild [2], such as the Australian zebra finch (*Taeniopygia castanotis*).

## 2. The zebra finch as a model songbird

The zebra finch is most prominent in the literature as the best-studied songbird and song system under captive conditions [30]. This is exemplified by the extensive literature on topics such as male song learning [31–35], song perception [36–38], song control and development [39,40] and female and male song preferences, as well as mate choice [41–46]. However, zebra finches became such a well-studied model species in captivity, not necessarily because they represent songbirds well [47] but because they are a popular pet species that has simple dietary requirements, is comparatively easy to breed and rear, has short generation times and allows the study of a wide range of biological questions, including various aspects of the male song system [40,48]. Furthermore, they breed opportunistically, with their gonadal maturity and spermatogenesis disassociated from seasonal cues such as day length [49]. Zebra finches presumably have these traits because they are adapted to the ecologically harsh and unpredictable arid Australian outback to which they are endemic [47].

However, despite the large body of literature addressing proximate aspects of zebra finch song [30,40], we still have a poor ultimate understanding of their song, i.e. why zebra finches sing, with only a few studies on their song in the wild [50–54], and most other knowledge from the wild consisting of primarily anecdotal information [55,56]. For example, little is known about the social context of mate choice and song learning in the wild [57]. Also, the facts that males primarily sing after pair formation, instead of before and during mate choice as many other species do, and that males sing throughout the year across various contexts [54], are not easily explained by common theory on song functioning in mate choice or territoriality. Moreover, while zebra finches are well-established as a social and nomadic species [55], recent work has highlighted the nature of individual connectivity in their socially complex multi-level societies [58–60], raising the question of the role of acoustic signals in regulating these dynamics.

This limited knowledge of zebra finch song in the wild thus contrasts sharply with the fact that our understanding of birdsong from a functional perspective under natural conditions comes from studies on wild songbirds inhabiting the Northern Hemisphere temperate zone [2]. These highly seasonally breeding species are commonly territorial during the breeding season and evolved under conditions with strong and relatively predictable seasonality that differ substantially from those of the zebra finch [40,47]. Zebra finches instead live in less predictable and highly fluctuating environments [61], form life-long pairs and selection most likely will act on optimizing reproductive success with the same partner over multiple successive broods rather than the typical single breeding event per season [24]. Thus, owing to differences in ecological context and social organization, extrapolating findings in captive zebra finches to wider ecologically relevant contexts of the well-studied territorial species in the Northern Hemisphere and *vice versa* needs to be done with care. Therefore, studies on the role of the song under natural conditions are helpful to ecologically and evolutionarily contextualize, as well as to generalize, the extensive knowledge from zebra finches in captivity. As such, the song of wild zebra finches provides a unique opportunity to understand the function of song in a species that is spatially and socially very differently organized from territorial songbirds, and to potentially broaden the view on functions of birdsong in general. To understand the function of song in the zebra finch in more detail, we here consider first the breeding ecology and social organization of the species, and thus the context in which the song has evolved and is used. Then we highlight the highly individualized aspect of their vocalizations in general, and song in particular, before discussing how this might impact their spatial organization. Within these contexts, we discuss insights from behavioural studies in the laboratory and integrate our recent work on zebra finch song in the wild.

## 3. Zebra finch breeding ecology and social organization

Zebra finches are the most abundant and widespread estrildid finch on the Australian mainland, occurring on grasslands of the hot arid and semi-arid zone, as well as grasslands and farms of the more temperate coastal regions [55]. They feed exclusively on grass seeds [62], and the availability of these grass seeds depends on the aseasonal and unpredictable rainfall that is a unique characteristic of Australia’s arid zone [61]. Consequently, zebra finches breed opportunistically, and when conditions are favourable they will keep breeding multiple times in succession, regardless of the season. Likewise, they may not breed for multiple years when conditions are too harsh [54] and/or predation pressure is extremely high [63]. This pattern of breeding is similar to many Australian bird species, with breeding periods typically lasting more than twice as long as breeding seasons in Northern Hemisphere temperate species [64]. As zebra finches mature rapidly, birds that hatch at the beginning of a breeding period may even reproduce later in that same period [55]. Thus, their breeding ecology and the selection on breeding decisions of a pair differ strikingly from those in temperate zone species that typically have a single brood in a breeding season before they breed again, usually with a different partner, in the following year (e.g. see fig. 1 in Ref. [24]).

Furthermore, in contrast to many species in the more seasonal temperate zones of the Northern Hemisphere, zebra finches mate for life and form pair bonds very early in life, likely around the time that they become sexually mature [65]. They are socially and sexually monogamous, with extra-pair paternity rates of only about 2% in wild populations [66,67]. In the wild, the main unit in which they are observed throughout the year is the pair [58,59,68]. Even during periods in which no breeding has occurred for months in the local area, and will not occur for many subsequent months, a male and female can be observed to move around very closely together [58,59,68].

Zebra finch pairs live in multi-level societies, with other pairs (and unpaired individuals) joining each other when foraging [59,69], drinking [68] or socializing [58]. Zebra finches are not territorial but breed in synchrony in loose colonies [55,60,70]. Although the potential advantages of this breeding synchrony have not been formally tested, they could include the presence of peers for their young (which affects their development [43] and allows the forming of crèches, which has only been anecdotally described [55], and lowering the chances of nest predation and post-fledging predation, which can be substantial [55,63]. To attain breeding synchrony the birds use social cues, inspecting conspecific nests and preferentially starting to build their own nest near conspecific nests in an early stage of breeding [70,71]. They furthermore appear to pay attention to acoustic cues from nestlings when judging the suitability of the breeding habitat [72]. Acoustic cues themselves also appear to influence the breeding phenology of zebra finches, at least in captivity. In a study on captive zebra finches, birds that received additional recorded sound from their own colony bred more synchronously than birds from a colony that did not receive sound supplements [73]. In the wild, song varies across the breeding period and likewise could play a role in synchronizing breeding activity at a broader scale, permitting a greater level of social integration [54].

## 4. Zebra finch vocalizations and their individual signature

Overall, vocal communication is a conspicuous aspect of zebra finch life as both sexes call almost constantly [55], and males also sing throughout the year [54]. Zebra finch males and females have a large call repertoire with 11 calls described [55,74], probably because of the wealth of social contexts that they experience. For example, when determining the suitability of a nest site, ‘ark’ calls are used [55], whereas at the nest, zebra finches produce soft calls to coordinate parental care such as turn-taking with their partner [75–77]. During take-off in a group they produce ‘stack’ calls [55]. Indeed, the call activity across different social contexts, often being finely timed in coordinating pair members, constitutes a core part of their communication [78–83].

Vocalizations also play an important role in establishing this pair bond, as zebra finch males accompany their courtship display by song [48]. Song in this context is referred to as a directed song. Yet, by far, the most song produced by males is after pair formation, where zebra finch males keep singing in various social contexts [54]. Songs produced outside of courtship contexts are so-called undirected songs [48,84], mainly because these songs do not elicit an obvious and apparent immediate response from others. There are subtle acoustical differences between undirected and directed song, with undirected song having fewer introductory notes, being sung slower than directed songs [85] and being more variable [86]. Nevertheless, given the high temporal resolution of their auditory system [36–38], it is not surprising that these differences are meaningful to zebra finches, with females preferring directed versus undirected song, in addition to preferring the song of their mate versus the song of an unfamiliar individual [87].

**Figure 1.**
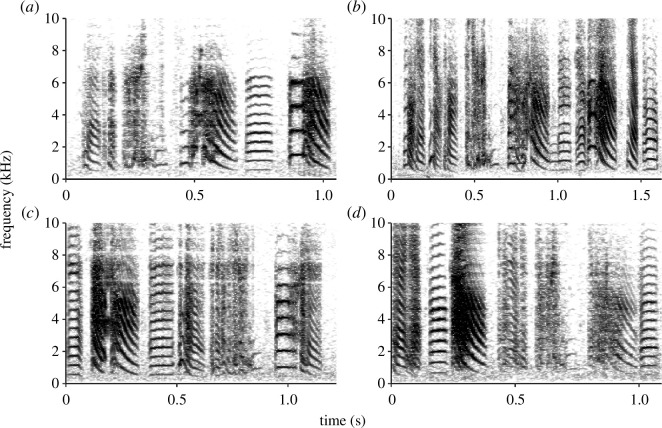
Sound spectrograms of four individually distinct song motifs, from four different wild zebra finch males. Spectrograms created in R using the ‘seewave' package, with a window length of 1024 and 80% window overlap.

As each male sings a more-or-less stereotypic, individually distinct motif [85] consisting of various classifiable element types [88], individual males can be readily recognized based on their song signature (figure 1) by human researchers, which has likely played a role in them becoming an important system for studying song learning. The song can thus be used for immediate individual identification by receivers [44,89]. Indeed, individual identification appears to be highly relevant in their communication, given that captive zebra finches are also able to recognize individuals across the other call types in their repertoire [83,90,91] and different levels of degradation [92]. However, these different calls and degradation levels require knowledge of call-specific cues to identify the signaller’s identity because there are no apparent overarching voice characteristics unique to an individual [83], as found in some other species of birds and mammals [93,94]. Although many of these individual recognition studies typically test for binary discrimination using unfamiliar versus familiar individuals, identification of a zebra finch in the wild will be more complex in the dynamic multi-level society in which individuals come and go and most likely differ more subtly in the degree of familiarity. Individual recognition thus will be facilitated by high vocal activity [54,55] as well as by the individually distinct songs [93,95] and striking auditory abilities [36–38] that we observe in zebra finches.

## 5. Zebra finch song as a within-group signal

Since zebra finches do not hold territories and primarily sing after pair formation, the selection pressures on song presumably are very different from classically studied songbirds in the temperate zones with seasonal mate attraction and territoriality. General birdsong theory, where birds sing to defend a territory and attract mates (and extra-pair mates) [2], would therefore predict that zebra finches should not sing much at all (given that they remain paired for life and have little to no extra-pair mating [55,67]), yet they do. Indeed, in a recent study that acoustically monitored a population during an extended period of drought which included a period of around 30 months with no breeding, song was recorded on a continuous basis throughout that non-breeding period, i.e. in 100% of 13 analyzed consecutive months [54]. Thus, while song is clearly linked to finding a mate during the rare periods in life when an individual is unpaired [50] and to breeding activity [51,54], these functions cannot explain most zebra finch singing activity. This being the case, why do zebra finches sing and what do we know from the field on the context of singing and its function in the social organization of the species?

Zebra finches exclusively sing very softly, with measurements from laboratory populations ranging from ±51 dB [96] to ±65 dB [97], whereas in the wild the loudest part of the song is produced at ±51 dB [53] (dB at 1 m for all values in this paper). Therefore, their song cannot function to regulate spacing over long distances as it does in territorial species in the temperate zone [4,98]. This is in stark contrast to other songbirds, which sing between 70 and 100 dB [17,99], or even louder in some suboscines [100] with the song often carrying over more than 100 m [14]. The distance over which zebra finch song can function is in fact extremely short, when considering also their hearing abilities. An integration of the natural singing amplitude, sound transmission experiments using this amplitude and lab-determined zebra finch hearing audiograms derived from pure tone discrimination [101] indicates that zebra finches can hear each other’s song over only about 9 m [53]. This limited distance even refers to only the loudest part of the song, so that they must be even closer to each other to be able to extract full information from more subtle features within the song. In line with this, in a laboratory study investigating whether zebra finch males adjust the amplitude of their song to the receiver distance, only 50% of males sang to females at a distance of 80 cm and only 20% of males sang for a female at 320 cm [102]. Also, the ‘distance call’—the loudest call in their repertoire—cannot be heard over much larger distances, so likewise cannot function to regulate spacing beyond those individuals already nearby in the same tree or individuals passing by in the vicinity [53]. Zebra finches are well able to discriminate among highly degraded vocalizations [92], yet they need to be close enough to be able to hear them. In combination with the frequent wide spacing of vegetation in their habitat in the Australian outback, the song can thus function to communicate towards members of the same group in the same or adjacent tree or bush, but not to regulate the spacing over larger distances much beyond their visual range, as in songbirds with loud territorial song.

Further field-based evidence that song is used as a within-pair or within-group signal is shown by Loning *et al*. [53], who observed during transect walks that singing males were typically in close physical proximity to a female (presumably their partner) or a larger group. Only in 12 of 94 observations (13%), singing males were observed singing alone, indicating that zebra finches most commonly, although not exclusively, sing when they are with others [53]. Moreover, adjacent groups were more than 20 m apart, so beyond the estimated hearing range of about 9 m for song and 16 m for the distance call, the loudest call in the zebra finch repertoire [53]. Thus, these transects and the calibrated field recordings provide evidence from different perspectives that song and calls are not used as long-range signals. That song can also attract other nearby individuals was found in another playback study, where song at nest boxes attracted other individuals, pairs and groups that were passing nearby [54].

These studies together thus show that song is a short-range signal to conspecifics that are already nearby and it attracts rather than repels others that pass by in the vicinity. Since the most common social unit in wild zebra finches is a pair or a social group [58], the short communication distance here may even select for singing softly although one cannot rule out a reversed causality, where their soft song leads to individuals seeking close proximity to gather socially relevant information. Such consistently soft song with a short communication range is, therefore, a clear departure from the traditionally assumed primary function of birdsong as a long-range advertisement signal used in territory defence and mate attraction [1,2,103]. This deviation from these classically sexually selected functions is similar to the way that female song has been demonstrated to be a clear departure from the classical view [21]. Following the approach of Odom *et al*. [21], it would be valuable to conduct comparative analyses to elucidate whether birdsong is ancestrally loud or soft, but data on vocalization amplitude in many more species would be required.

## 6. The indirect role of song in spatial organization of zebra finches

While sound in many species is key to regulating spacing [4,12], it thus appears that softly vocalizing species like the zebra finch must have evolved other mechanisms to regulate their spacing over larger distances, specifically to find each other beyond the visual range once they lose close contact. During breeding times, one mechanism is to scan for conspecific nests, and as zebra finches often breed in loose colonies, nests will be close to each other and contact will likely be re-established with other conspecifics. Indeed, zebra finches regularly inspect each other’s nests [104], which facilitates synchronized breeding. Moreover, males sing at the nest and singing activity varies with the nesting stage [51,54]. As a consequence, neighbours and birds passing through an area can relatively efficiently gather information on individuals present and their breeding stage by visiting nests and attending to song [54]. However, as their soft song does not act as a long-range signal, it cannot play a significant role in effecting social connectivity by attracting others over longer distances. This is especially the case outside of the breeding context, when there is no clear neighbourhood structure with fixed nest locations. Thus, we suggest that the social cohesion of multi-level societies of zebra finches that persists outside the breeding season, where birds move around in pairs and then meet others repeatedly, is facilitated by the combination of movement routines and frequent individually distinct singing and calling. One option to find others surely is the limited number of places to find water, so approaching a water source will almost certainly lead to social group formation, either at the water itself or along commonly used flyways [69]. However, birds can also be seen in small groups away from water [53,54,68], so that it is likely that other spatial mechanisms play a role.

One of these mechanisms may be the attendance at social gathering sites. It was recently revealed that zebra finches use social hotspots, which are trees or bushes at which they hang out during the day [58]. These hotspots are used repeatedly over longer time periods (several months or years), so appear to be learned or culturally transmitted sites for socializing. These gathering sites are interesting as they are not primarily a mechanism to gather and then move on as a group, but birds arrive and depart mainly in pairs [58]. Since in addition to actively calling, males regularly sing at these hangouts, the function of song can be expected to have wider social functions and implications. During these social gatherings, males may use song to establish and maintain individual social relations with others, and repeated singing may facilitate individual recognition in a dynamic social environment with many singers [95]. Specifically, the prominent individual distinctiveness of the zebra finch song is likely to be a key component to maintain social connectivity because individuals will be able to readily identify familiar individuals near their nests or in social hotspots [54]. The individual song signature thus may be a driving mechanism to also influence movement patterns in ways that are very different from territorial songbirds or animals with long-range signals in general.

**Figure 2.**
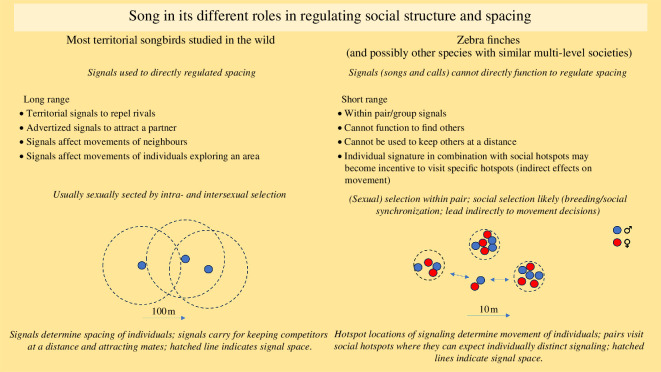
Schematic overview of the function of long-range signals such as song in the classical advertisement and territorial context and the dynamic social context as seen in zebra finches.

The singing (and calling) at social hotspots in zebra finches, which cannot function in attracting or repelling individuals over distance, instead may create a spatio-social structure that facilitates movements to these places at certain times of the day because birds that visit those hotspots can expect to find others singing, including known individuals (figure 2). Future work is thus needed to determine the vocal behaviour at the hotspots, taking into account singing and calling, the latter of which has been shown to be highly coordinated among pairs and within groups [77,82,105]. Birds having individual experiences or relations with particular other individuals may thus seek to ‘hang out’ in those places at which they can expect to encounter specific other individuals. The individual identity of the song (and distance calls in males and females) then may help in tracking dispersal or survival in the population because changes in the acoustic environment at the social hotspots would provide information on immigration and of individuals dispersing or dying. This could act as a proxy for survival conditions and predation pressure [63]. An interesting parallel to these ideas relating to the individually distinct song of the zebra finch is the individually distinct facial plumage patterns described in the red-billed quelea *Quelea quelea* [106], which is ecologically similar to the zebra finch, with birds ranging over sub-Saharan Africa in large flocks that breed colonially and follow quite a nomadic lifestyle. In this species, the individually distinct facial plumages lack condition dependence and may instead function in facilitating individual interactions by allowing individual recognition in a species with a rich social environment [107].

The social hotspots that were recently described in the wild zebra finch [58] held birds for significant parts of the day (36% of the daylight hours) and acoustic activity was recorded for a high proportion of the time that birds were present at the hotspot. As such, there is potentially a lot of information that individuals can exchange while they are spending time with conspecifics in this context. As these social hotspots have only recently been characterized, no work has yet examined the type of variation in acoustic behaviour that may be present in such contexts across different ecological conditions, with respect to the social mix of individuals present, how regularly individuals spend time at these locations and if the same set of individuals meets at these sites repeatedly. Furthermore, if individuals are heard singing regularly over time, then potentially there will be pertinent information within the variation in song performance that provides insight into an individual’s state (see [96,108]). In the future, it will be interesting to determine whether and to what extent the individual singing activity or other aspects of song performance, such as motif repetition pattern and subtle features in song production, specifically within and between known individuals at these hotspots, provide relevant information on condition [96,109], environmental conditions [110], foraging efficiency [108] or breeding state [54].

## 7. Conclusions

Given the nature of singing in wild zebra finches, it would be interesting to imagine how the birdsong literature would have developed if research had started by studying zebra finches in the wild instead of territorial songbirds in temperate regions or zebra finches in captivity. We probably would have initially developed a different and less dualistic view on the function of birdsong. Based on the current evidence from the wild, it is clear that zebra finches are different in their song behaviour compared with those typically studied songbirds in the wild, e.g. tits, flycatchers and song sparrows [2,6,7,10] because zebra finches primarily sing outside the classical contexts of mate attraction and territoriality. Instead, they sing their individually distinct song motif all year round in a range of social contexts, when alone with their mate and in larger social settings. This singing behaviour makes a fascinating case for the wider role of individually distinct vocalizations in the social organization of animal societies and widens the required considerations to understand the evolution of birdsong and animal communication, to be more integrative across a wider range of contexts and selection pressures. It will be interesting to see in the future if mated females attend to variation in their mate’s song in the same way as they do when tested in a mate choice paradigm [84] because sexual and social selection after mate choice, rather than during mate choice, might pose the strongest selection pressure on their singing [24].

Such a social function of song, and particularly within the partnership, is consistent with the short audible range of zebra finch song of just a few metres. The softness of the song will therefore not directly influence movements across large distances. Yet, the singing at specific social hotspots will likely affect movement indirectly, as birds may specifically visit those hotspots to obtain auditory information from others. The individually distinct song, for instance, can provide information on population changes, such as the appearance of new individuals and the disappearance of known individuals. It thus may allow birds to estimate survival and predation threats, as well as functioning in breeding synchronization given that males sing more in groups during breeding. Singing all year round may also be part of staying in latent breeding conditions in an environment where conditions may change unexpectedly. Singing thus may be essential to maintain a closer partnership and to keep the vocal apparatus in shape. Recently, Adam *et al*. [96] showed that regular singing practice is essential to keep the motor apparatus in shape and that females pay attention to such relatively subtle differences. The perception of this subtle variation in song [96], also suggests that perhaps both males and females can receive valuable information from the population of individuals singing in the social contexts that we have described.

Taken together, the research on zebra finches in the wild broadens our view on the role of signalling and birdsong, in particular, for the regulation of spacing and social organization, as developed from studies on territorial species with loud advertisement signals. By integrating social hotspots, spatial routines and signalling, our studies suggest that signalling with individually distinct signals at specific sites can determine movements in an indirect way, as others may visit these sites specifically with the expectation of gathering information rather than being attracted directly by signals from the distance. To understand the role of signals in social organization, the integration of research on communication and movement across different social systems thus can reveal exciting and very different angles on how animal societies function.

## Data Availability

This article has no additional data.
